# IncobotulinumtoxinA Injection to Balance Eyebrows and Facial Shapes: A Review with Illustrative Clinical Examples

**DOI:** 10.3390/toxins18070296

**Published:** 2026-07-09

**Authors:** Carla de Sanctis Pecora, Birgit Blessmann-Gurk, Bianca Viscomi

**Affiliations:** 1 Private Practice, Sao Paulo 04548-004, Brazil; 2 Private Practice, 20354 Hambrug, Germany; dr.bbg0815@googlemail.com; 3 Private Practice at Bianca Viscomi-Clínica de Dermatologia, Sao Paulo 01433-010, Brazil; biancaviscomi@yahoo.com.br

**Keywords:** face, eyebrow, botulinum toxin A, incobotulinumtoxinA, neurotoxin, injection technique, facial balance, optical illusion

## Abstract

The eyebrows are a central element of facial harmony, influenced by individual and external factors such as gender, ethnicity, facial shape, age, culture, and fashion trends. Traditional methods for esthetically enhancing eyebrows include plucking, highlighting, and surgical interventions. Another minimally invasive option for altering, thus enhancing eyebrow positioning, is botulinum toxin type A (BoNT-A) injection. The objective was to demonstrate a systematic, customizable approach for BoNT-A injections to reshape and reposition eyebrows according to individual facial shape enhancing balance. To determine optimal BoNT-A dose and injection points, an individualized assessment of facial and eyebrow shape and position was performed, leading to a customized protocol of neurotoxin injections according to each patient’s needs. The assessment method aims for the best match between the individual facial shape and eyebrow form and position. This technique enables individualized modulation of eyebrow position and shape, improving facial balance while addressing dynamic upper facial rhytides, thereby promoting harmonious outcomes that preserve individual facial characteristics and providing an additional approach to conventional eyebrow enhancement techniques.

## 1. Introduction

The eyebrows have been recognized as a central element for communication, personality, facial expression and harmony. There is no singular “ideal” eyebrow shape universally applicable to all faces. Age, gender, culture, ethnicity, facial shape, and fashion trends influence the concept of the ideal brow [1,2]. While interfering with the eyebrow features, it is important to have in mind that the perfect match between facial shape and eyebrow design and position is key to improving balance, aiming for an ideal harmonious facial frame [3,4].

Historically, women have been seeking treatments that could improve facial harmony by interfering with the eyebrow look, exploring techniques such as plucking, painting, trimming, and highlighting, and more recently, micropigmentation or permanent makeup, even surgical interventions have taken place.

Moreover, after the emergence of botulinum toxin type A (BoNT-A), injectors observed that by blocking mainly the medial and lateral brow depressor muscles, the eyebrow position could change, using a minimally invasive and safe procedure [5,6]. Carruthers et al. [7] demonstrated that selective neurotoxin-induced blockage of the inferomedial fibers of the frontalis muscle leads to a compensatory increase in resting tone of the untreated frontalis fibers, resulting in elevation of the eyebrow.

Despite the generally favorable outcomes of neurotoxin injections in the upper third of the face, suboptimal injection techniques may result in undesirable esthetic outcomes, including an unnatural or frozen appearance, eyebrow ptosis, and brow asymmetry. These complications are particularly relevant in female patients with low-set and flat eyebrows. Hence, there is a need for a customizable treatment to shape and position the eyebrows that aligns with individual patient preferences while ensuring suitability across diverse facial morphologies, thereby enhancing overall facial harmony [8]. On this basis, the objective of this research was to structure a systematic, customizable approach for BoNT-A injections to reshape and reposition eyebrows according to individual facial shape, enhancing balance.

## 2. Results

### 2.1. Search Results and Trial Characteristics

Initially, the literature search yielded 80 studies, from which 34 duplicates were removed. Fourteen studies were included in the review.

### 2.2. Eyebrow Shape and Position

The eyebrows play a crucial role in facial identity and emotional expression; therefore, alterations in their morphology or position may disrupt the holistic perception of the face and potentially result in misinterpretation of emotional cues [9,10]. Their position results from the anatomical structure of the upper face and the dynamic balance among the frontalis muscle—the only elevator of the upper face—medial depressors (corrugator m., procerus m. and depressor supercilii m.) and lateral depressors (orbicularis m.) [11]. There is a great variety in the size, shape, and position of eyebrows that are esthetically pleasing on different faces. Here, we describe five main brow shapes referred to as: (1) curved, (2) sharp-angled, (3) soft-angled, (4) rounded, and (5) flat. Brow-arched configurations may be further classified into low, medium, and high arches. Collectively, these eight primary brow characteristics can be modified to optimize facial balance according to individual face shape [12].

### 2.3. Face and Eyebrow Shapes and Their Importance for Facial Balance

Neglecting the esthetic relationship between eyebrow shape, position, and overall facial morphology leads to an oversimplified, uniform approach to facial treatment, thereby disregarding individual facial characteristics. In practice, the ideal eyebrow shape, length, and position should be tailored to each facial type to preserve balance, harmony, and facial identity.

There are six primary facial shapes: oval, round, square, heart, and long. The oval face is often regarded as the “ideal” shape being considered the most balanced one. Therefore, shaping the eyebrow design of oval face individuals can be chosen based on personal preferences and trends. The objective of interfering with the eyebrow shape and positioning, according to individual’s facial shape, is to improve facial balance and enhance beauty. For instance, long facial types should benefit from flatter eyebrow design, which generates horizontal visual vectors that promote facial harmony and modulate overall perception. In contrast, arched eyebrows create vertical visual vectors that accentuate facial length, making this configuration more suitable for round facial shape [9,13].

Recommendations for ideal eyebrow morphology according to facial shape, including suggested adjustments in the eyebrow length and position, are summarized in Table 1 [12].

Determining face shape can be challenging during assessment. Therefore, placing a transparent sheet over a photograph of the face, followed by tracing the facial contour [14], may be helpful.

### 2.4. Gender Differences Regarding Eyebrow Position

The appearance of the upper face and eyebrow is also influenced by gender. Sexual dimorphism in the facial skeleton contributes to distinct structural characteristics between males and females. Men typically exhibit greater height and width in the forehead, with less convexity and more of a backward slope to the hairline [15]. Studies showed that the average forehead height is approximately 5 cm in women compared to 6 cm in men [16]. Furthermore, men display more pronounced supraorbital ridges that merge with the glabella, resulting in a more prominent glabellar area, interfering in the eyebrow position and shape.

The male brow is generally thicker, heavier, and flattened, whereas the feminine brow tends to be more curved and peaks at the junction between the middle and lateral third of the brow [17]. Additionally, the vertical distance from the brow to the eye is shorter in men, as the brow tends to rest above the supraorbital rim with minimal arch [18]. These key distinctions are crucial to account for in facial beautification and rejuvenation treatments, having in mind gender identification. It has also been suggested that aging impacts male and female esthetics differently; for instance, the female aging process tends to have a flattening of the forehead and a more prominent supraorbital bridge. Moreover, although there are differences between genders, women’s eyebrow shapes are frequently modified in response to fashion trends, whereas men’s tend to remain relatively stable. Nevertheless, the growing interest in cosmetic procedures among men could introduce new trends, like those seen in women [18].

### 2.5. Botulinum Toxin Injections to Change Eyebrows and Improve Facial Balance

BoNT-A was approved for esthetic purposes in 2002, initially for treating glabellar wrinkles [19] and afterwards, for upper face treatments, including forehead and crow’s feet [20].

Over time, its uses have expanded to treat masseteric hypertrophy, gummy smiles [21], skin quality [22,23], chronic sialorrhea [24,25] and hyperhidrosis [26], as well as for neurotoxin injections in the inferior third of the face, for eyebrow reshaping and repositioning, the halo of action size of the BoNT-A brand, accuracy, and the precision of the injection technique could affect the results [27]. Therefore, awareness of the volume of saline solution used for the vial reconstitution and the halo of action size for each neurotoxin brand is essential when injecting muscles of facial expression to appropriately modulate muscular balance [5,27]. It has been demonstrated that IncoBoNT-A has a precise halo of action comparable to that of OnaBoNT-A, as shown in a study comparing the anhidrotic halos produced by three different botulinum toxin formulations following intramuscular injections [28]. In a non-comparative study design, Mariwalla et al. demonstrated that the clinical benefits of DaxibotulinumtoxinA injection for the treatment of dynamic glabellar wrinkles are beyond effacement of lines, showing smaller diffusion and predictability in miomodulation, favoring more consistent outcomes while balancing frontalis and brow depressors, and consequently, eyebrow dynamics [29]. Reconstitution volume and dosage per injection point can also interfere with the spread of the neurotoxin injection; the higher the volume and the dosage, the larger the spread of the toxin [25].

Positive effects of BoNT-A injection include correction of brow asymmetry, improvement of brow shape, and raising or reshaping of brows that have changed as a result of age-related process [10]. Eyebrow position and shape can be modified through neurotoxin injection by modulating the muscular balance between the occipitofrontalis and antagonistic muscles, medical and lateral brow depressors. Occipitofrontalis is the only levator muscle in the upper face in opposition to the brow depressor complex, including the corrugator supercilii, the procerus, depressor supercilii, and the orbicularis oculi [11,30]. The objective of the treatment goes beyond reducing dynamic lines; it is to selectively weaken the frontalis m. and the depressor complex, rebalancing the brow muscles to obtain an esthetically pleasing eyebrow position and shape [7,11]. Accurate determination of frontalis injection sites requires an understanding of the principle that partial chemodenervation of a muscle increases the resting tone of the remaining untreated fibers, thereby altering intramuscular force balance. Consequently, selective blockade of the central fibers of the frontalis muscle, while sparing the lateral fibers, results in a relative increase in resting tone and functional dominance of the untreated lateral fibers [7,11]. Therefore, to enable a detailed assessment of the frontalis muscle strength distribution pattern, we recommend the use of the ONE21 technique [8].

**Figure 1:**
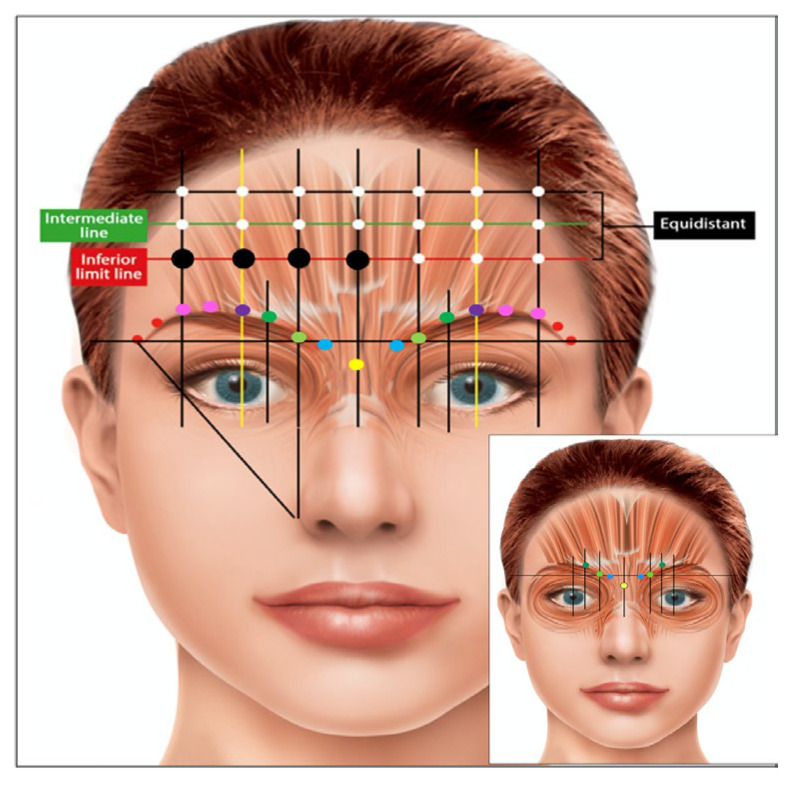
Schematic lines on the face for planning the points of injection. Miniature shows only glabellar injection sites and periocular muscles. Colored circles and segments represent the location of injection points: light green and leaf green dots represent the medial and lateral corrugator supercilii, respectively. Yellow dot represents the procerus injection point (a second injection point can be added 1cm above, up to the intersection point with the glabellar line for stronger muscle). Blue dots: depressor supercilia. Red and pink dots: orbicularis superior and lateral superior, respectively. Black dots: frontalis m. Purple dots: when there is a combined action of the orbicularis with the lateral end of the corrugator. White and black dots in forehead: possible points of injection. White dots: block the movement and treat the wrinkles. Black dots: treat inferior wrinkles when present and or reshape the eyebrow.

For the assessment of the forehead dynamic wrinkles, seven vertical lines can be drawn based on the inner canthus line, outer canthus line, mid-pupillary line (MPl) and mid-facial line. Then, the functional horizontal lines, the lowest horizontal frown line, the uppermost contraction line, and a third one equidistant from the two lines are defined. The intersection points of the vertical and horizontal lines in the forehead are possible injection sites for the treatment of the forehead dynamic lines—white and black dots [31] (Figure 1).

Treatment of the medial brow depressor muscles is key to rebalancing opposite muscular forces and should be guided by a detailed and individualized assessment of glabellar dynamic wrinkles pattern, as well as by a targeted injection technique that respects the anatomical position and depth of the muscles in this region. The corrugator supercilii muscle originates from the superciliary arch in the paramedian plane and becomes progressively more superficial as it extends laterally and cranially, blending with the orbicularis oculi muscle within the medial eyebrow region before inserting into the dermis. The procerus muscle arises from the nasal bone at the nasal root, lying deep to the orbicularis oculi and within the same anatomical plane as the corrugator origin, before inserting into the glabellar skin near the superior eyebrow margin. Among the brow depressor muscles, the depressor supercilii is the most superficial [32]. Insufficient understanding of the regional anatomy and inaccurate injection techniques may block undesired muscle fibers, jeopardizing the outcome [8,31].

For this purpose, an additional horizontal line based just below the inferior limit of the eyebrows and two other vertical lines can be marked between the inner canthus line and the MPL to complete the anatomical landmarks guiding the placement suitable injection points for the glabellar wrinkles treatment, always respecting a distance of 1 cm from the orbital rim [8]. Assessment of dynamic glabellar lines is based on the premise that every dynamic wrinkle is formed perpendicularly to the muscle fibers. Accordingly, a horizontal line at the nose’s root reflects contraction of the procerus, whereas vertical lines in the central part of the glabella are a consequence of the contraction of the corrugators [8,32]. A curved line in the medial inferior end of the eyebrows indicates a contraction of the depressor supercilii, and when there is a combined action of the orbicularis with the lateral fibers of the corrugator, some small parallel lines can be seen in the medial third over the eyebrow. In the central part of the forehead, the visualization of horizontal parallel lines will suggest the combined action of the frontalis. The accuracy of the glabellar injection sites for botulinum toxin type A injection is also key and must respect the layered muscle anatomy, which includes superficial injections to the lateral corrugator, which overlies the frontalis; deep injections to the inferior medial corrugator, in line with the inner canthus, which underlies the frontalis and where the bulk of medial corrugator mass is located for most patients; and deep injections to the lower procerus, where the bulk of the muscle mass is located, at the level of the selium. Avoid high procerus injections above the glabellar line, where the frontalis interdigitates with the procerus, thus weakening central frontalis activity [8,33,34].

Following this analysis, neurotoxin injection planning can be undertaken to change the eyebrow shape. In the frontalis muscle, the intersection points of the vertical and horizontal reference lines along the inferior limit line (black dots)—the lowest horizontal line in the forehead—serve as a guide for evaluating eyebrow shape and for determining appropriate injection sites (Figure 1) [31].

Achievement of an eyebrow arching effect requires targeted modulation of the frontalis muscle, as well as the medial and lateral brow depressors, tailored to the patient’s original eyebrow morphology and clinical-anatomical pattern. In a nutshell, intersection point between the medial facial line and the inner canthus line with the inferior limit line of the forehead is injected, avoiding injection of the lateral ones. It increases the resting tone of the lateral inferior, unblocked or partially blocked, resulting in a lifting of the lateral part of the eyebrow. Use the lowest dosage in the inferior horizontal line (black dots), as this part of the frontalis, is mainly responsible for raising the eyebrow [8,11].

**Figure 2:**
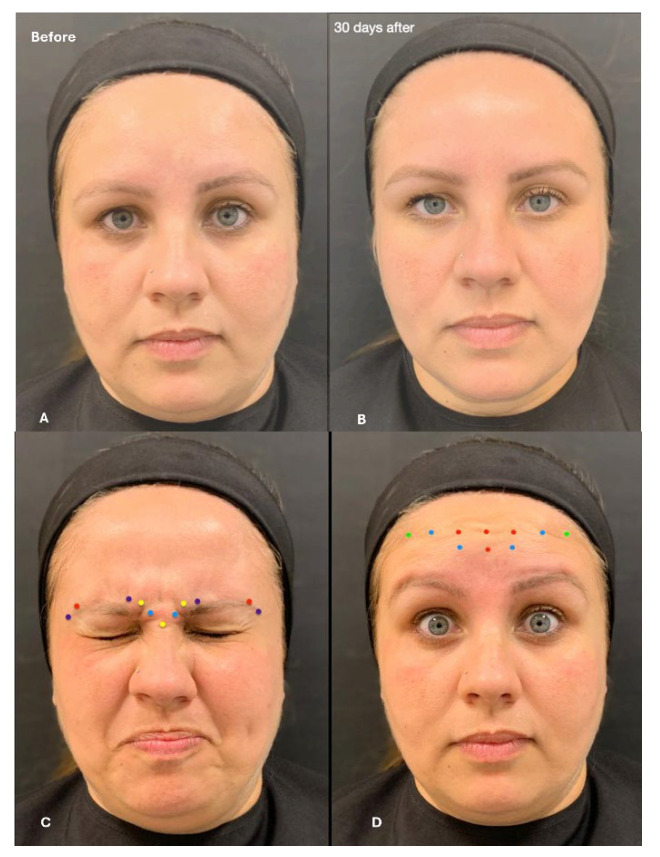
(**A**): Example of a round face with flat eyebrows, before treatment. (**B**): Same face 30 days after treatment. (**C**): Injection points with respective BoNT-A dose for the treatment of the glabellar line complex. (**D**): Injection points with respective BoNT-A dose for the treatment of the horizontal forehead lines. Doses of IncobotulinumtoxinA (Merz Pharmaceuticals GmbH, Frankfurt, Germany): green dots: 0.5 U; blue dots: 1 U; red dots: 2 U; purple dots: 3 U; and yellow dots: 4 U.

**Figure 3:**
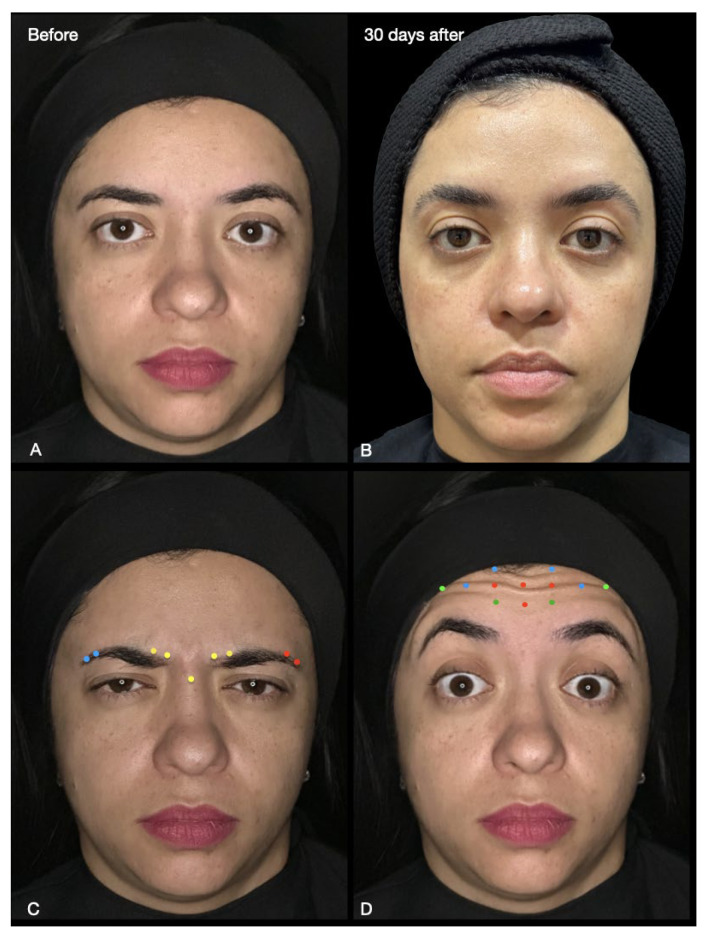
(**A**) Example of a heart-shaped face with a small asymmetry in eyebrow shape (right brow flatter than the left one), both presenting the lateral end of the eyebrow lowered positioned in the horizontal line while compared with the medial end, before treatment. (**B**) Same face at rest 30 days after treatment. (**C**) Injection points with respective BoNT-A dose for the treatment of the glabellar line complex. (**D**) Injection points with respective BoNT-A dose for the treatment of the horizontal forehead lines. Doses of IncobotulinumtoxinA: green dots: 0.5 U; blue dots: 1 U; red dots: 2 U; yellow dots: 4 U.

**Figure 4:**
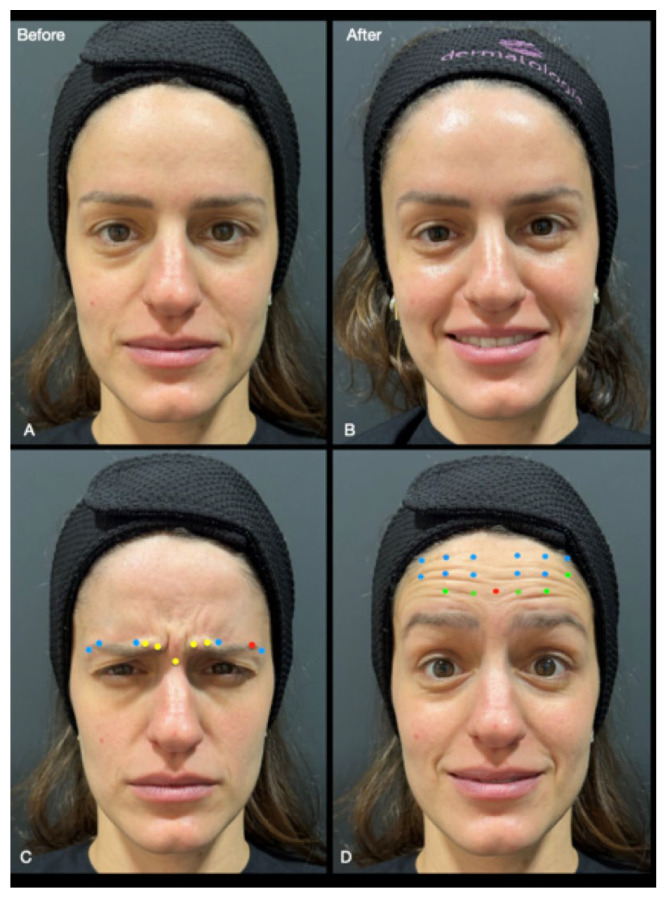
(**A**) A patient with a long-shaped face before the treatment, presenting with naturally flat eyebrows, a brow configuration that contributes to facial balance by emphasizing horizontal facial proportions. (**B**) The same patient at rest 15 days after treatment. (**C**) Injection points with respective BoNT-A dose for the treatment of the medial and lateral brow depressors. (**D**) Injection points with the corresponding BoNT-A dose for the treatment of the horizontal forehead lines. Doses of IncobotulinumtoxinA: green dots: 0.5 U; blue dots: 1 U; red dots: 2 U; yellow dots: 4 U.

If the objective is to flatten the eyebrow, injection in the intersection points of the outer canthus line and MPL with the inferior limit line should be performed, using a higher dosage in the MPL intersection point [31,35]. For those subjects with naturally arched eyebrows, apply a higher dosage along the lateral vertical lines (outer canthus and MPL) and a lower dosage in the central area. On the other hand, for those with natural flattened eyebrows, maintain an equal dosage in the entire inferior limit line, both laterally and medially. To drop the lateral end of the eyebrow, inject in the outer canthus point of the inferior limit line, creating a decreasing gradient of dosage towards the medial forehead [31,35]. Nevertheless, if the objective is to maintain the original eyebrow shape, the inferior limit line should be left without any injection or injected with the smallest amount necessary to erase inferior contraction lines, using the same dosage in the whole inferior line, to block it partially, avoiding eyebrow ptosis [8]. Detailed recommendations regarding injection points and dosage can be found in Table 2 and Table 3. Examples of treatment carried out in three female patients, illustrating the alignment between facial shape and the ideal eyebrow shape and position, together with the related scheme of injection sites and dosages of IncoBoTN-A are shown in Figure 2, Figure 3 and Figure 4.

Repeated contraction of the facial expression muscles involved in smiling and squinting, particularly the orbicularis oculi muscle, contributes to the development of lateral canthal rhytides (crow’s feet lines). The orbital portion of the orbicularis oculi muscle, responsible for voluntary eyelid closure and eyebrow protrusion, plays a central role in the formation of crow’s feet lines, and repeated contraction may lead to lateral eyebrow ptosis. Treatment of the superior lateral aspect of the orbicularis can be made for the lateral eyebrow lifting, injecting one or two points of 1, 2, and 3 U in the superior lateral fibers of the orbicularis oculi, respecting the distance of 1 cm from the orbital rim [11,36].

The muscular balance of the upper third of the face, particularly the functional interplay among the frontalis, corrugator supercilii, procerus, and orbicularis oculi muscles, is a key determinant of esthetic outcomes. Consequently, excessive treatment of the brow depressor muscles may result in an excessively elevated or overly arched eyebrow, whereas excessive weakening of the frontalis muscle may lead to eyebrow ptosis [37,38].

Nevertheless, studies in the literature reinforce the importance of treatment individualization, detailed anatomical assessment, and a comprehensive understanding of the dynamic balance between the eyebrow elevator and depressor muscles. Emphasis is placed on respecting functional anatomy and adapting injection points, depth, and dosing according to each patient’s specific muscular characteristics and esthetic needs. Furthermore, these findings have helped consolidate the concept that neurotoxin treatment of the upper face influences not only wrinkle reduction, but also eyebrow shape and positioning, representing an extremely important factor to be considered during the treatment of dynamic upper facial rhytids [37,38].

### 2.6. Eyebrow Asymmetry

A thorough evaluation of eyebrow shape and position, both at rest and during maximal contraction, is essential before neurotoxin treatment of upper face dynamic wrinkles, particularly forehead lines, allowing for proper alignment of patient expectations and individualized therapeutic planning. Pre-existing eyebrow asymmetries may arise from differences in underlying skeletal anatomy, variations in eyebrow hair positioning, and unequal functional activity between frontalis muscle bands secondary to habitual muscular use. Once the underlying etiology is identified, eyebrow balance may be optimized through modulation of the functional interplay between the frontalis and brow depressor muscles, using a strategic distribution of injection points and dose adjustment according to local muscle strength [39].

Dynamic palpation of the forehead during contraction is recommended to identify areas of increased muscular recruitment, where higher doses may be required. In cases of asymmetry induced by botulinum toxin treatment, reassessment of eyebrow position during maximal frontalis contraction, as well as evaluation of orbicularis oculi activity—the primary lateral brow depressor—is recommended to identify residual asymmetric muscle activity. In such cases, the administration of an additional 1–2 units of neurotoxin into the superior lateral fibers of the orbicularis oculi on the side of the depressed eyebrow may promote eyebrow elevation and repositioning [39].

### 2.7. Rationale for Rounded Face

Choice of injection points and dosage for the treatment of the frontalis aims at increasing the resting tone of the lateral inferior fibers that are left untreated. These combined treatment schemes resulted in the lateral arching effect of the eyebrow. By arching the eyebrow laterally, imaginary vertical lines are built in the round face, creating an optical illusion of a longer face while compared to the before picture, improving facial balance as shown in Figure 2.

### 2.8. Rationale for Heart-Shaped Face

Individualized injection strategy for eyebrow reshaping in a patient with a heart-shaped face. Frontalis injection points and doses were selected to increase the resting tone of the untreated inferior lateral frontalis fibers, producing a rounded, arched eyebrow that improved facial balance and softened the facial contours.

Dynamic assessment revealed greater frontalis activity on the right side, with a higher medial and lateral brow position during maximal contraction. The most pronounced asymmetry was observed at the mid-pupillary line (MPL), where the right frontalis demonstrated greater contractile strength. Accordingly, the MPL point was treated with 1 U on the right (blue) and 0.5 U on the left (green), resulting in a more balanced and symmetrical eyebrow position.

Different doses were also administered to the superior lateral fibers of the orbicularis oculi to correct the pre-existing eyebrow asymmetry. Two points of 1 U were injected on the right side, whereas two points of 2 U were injected on the left (Figure 3).

### 2.9. Rationale for Long-Shaped Face

An individualized injection strategy was designed to preserve the patient’s naturally flat eyebrow shape, which is considered the most harmonious configuration for a long-shaped face, while avoiding eyebrow ptosis. In Figure 4, we showed the frontalis injection points and doses that were selected according to muscle contraction strength, as assessed along the intermediate and upper horizontal contraction lines, to effectively reduce muscle activity responsible for forehead rhytides. Along the inferior horizontal line, 2 U (red) was injected at the central frontalis point, while four additional points located at the intersections with the inner canthus and mid-pupillary lines received 0.5 U each (green). This differential dosing strategy preserved the flat eyebrow contour while subtly elevating the entire eyebrow through a medial-to-lateral dose gradient. To achieve a slight elevation of the lateral brow tail without increasing the eyebrow arch, the inferior lateral frontalis fibers were intentionally left untreated, and two injection points were placed in the superior lateral fibers of the orbicularis oculi muscle on each side.

## 3. Discussion

The esthetic importance of the eyebrow has been highlighted for centuries. Across civilizations, beginning with ancient Egyptian and Greek societies, cosmetic practices such as the use of make-up have been employed to modify the eyebrow shape in order to enhance facial balance and harmony [12]. The eyebrow is an important feature of feminine beauty, being even celebrated in verse by the French poet Maurice Scève [40] who wrote the poem “*Blason du Sourcil*,” in the 16th century: “*…Sourcil qui fait moon espoir prospérer. Et tout à coup me fait désespérer. Sourcil sur qui amour prit le portrait. Et le patron de son arc, qui attrait. Hommes et Dieux à son obéissance, …*”. Scève described the beauty and expressiveness of women’s eyebrows, highlighting how they can convey a wide range of emotions and add a touch of elegance to the face. He praises the ability of eyebrows to communicate feelings such as courage, fear, joy, and sadness, demonstrating the importance of this feature in esthetics and non-verbal communication.

The human visual system perceives and recognizes faces through holistic processing that integrates facial features and their spatial relationships into a global representation of the face [13]. Considering that eyebrows have a significant impact on face identification, in 2015, Matsushita et al. [13] investigated how the eyebrow position affects the perceived shape and size of eyes. The authors suggested that face perception relies on holistic processing, where the characteristic of one facial element affects the perception of others, such as altering eyebrow positions can change the perceived shape and size of eyes. Similarly, in the same year, Morikawa et al. [9] made an experiment to understand how eyebrow position, eye shadow application, and viewing distance affect the perceived size of eyes. Their findings also highlighted the impact of eyebrows on the perception of eye size and their potential implications for facial attractiveness and makeup application.

For facial plastic surgeons who perform eyebrow lifting procedures, the interplay between the shape of the face, the eye, and the brow must be clearly understood. It must be a subject of discussion with the patient what changes truly enhance the beauty of the face, independent of fashion. Alex [12] highlighted the importance of the individualized facial analysis to balance and harmonize each person’s distinctive facial characteristics, emphasizing that the ideal shape, length, and position of the brow must be appropriately altered to fit different facial shapes [12].

Injection of botulinum toxin type A into the upper third of the face alters the balance among the facial expression muscles and, consequently, influences eyebrow position. Careful consideration of facial features and proportions is essential, as no one-size-fits-all approach is applicable. Injection sites should therefore be individualized according to each individual’s facial proportions, morphology, and specific characteristics.

As eyebrow position and shape are directly influenced by the balance between the frontalis muscle and the medial and lateral brow depressor muscles, a thorough understanding of the function and contraction vectors of each involved muscle is of paramount importance to ensure that botulinum toxin injections are accurately targeted to the intended anatomical points. In a publication by Nabil Fakih, an IncobotulinumtoxinA injection technique was described, emphasizing the importance of the combined blockade of the depressor supercilii, corrugator supercilii, orbicularis oculi muscles (including the superior and lateral portions), and frontalis muscle to achieve a brow-lifting effect in patients presenting with an omega-type glabellar contraction pattern [41]. These findings further highlight the importance of identifying the specific muscles involved in each individual’s facial expression dynamics.

The treatment of the dynamic forehead lines, with neurotoxin injection, is one of the most challenging techniques and must be performed cautiously [13,42]. To optimize treatment efficacy while preserving eyebrow position and morphology, the highest dosage of neurotoxin should be injected in the intermediate line, usually coincident with the line of convergence (C-line), and in the utmost contraction line. The lowest continuous horizontal forehead line (inferior limit line) may be injected either to treat inferior dynamic forehead lines or to modify eyebrow contour. In these cases, lower doses are administered along this line to preserve partial muscular activity and optimize esthetic outcomes, thereby minimizing the risk of an overly rigid or “frozen” appearance [35].

It is important to understand the halo of action of the neurotoxin and use the smallest volume possible for reconstitution, thus making the injection more foreseeable and controlled. The injection depth is intramuscular for utmost precision and efficacy. Another crucial aspect to consider is the correct injection points in “inferior limit line” of the forehead for eyebrow shaping. Injection sites medial to the mid-pupillary line are generally associated with the creation of an arch and lift of the eyebrow laterally. A flatter eyebrow configuration is typically observed when injection points in the inferior limit line intersect the mid-pupillary line, whereas injections placed lateral to the mid-pupillary line tend to induce a relative descent of the lateral eyebrow tail. For a soft arching effect, the administration of higher dosages medially and lower doses laterally is recommended. In addition to these recommendations, targeted treatment of the brow depressor muscles is essential when applying this technique to restore muscular balance and facilitate eyebrow elevation. The lateral eyebrow lacks direct bony attachment; therefore its position is strongly affected by the dynamic balance between the elevating forces of the frontalis muscle and the orbicularis oculi. The medial end of the eyebrow is firmly attached to the underlying layers, making the lifting effect of this portion more difficult [10].

Injection technique, particularly for the treatment of the medial brow depressors, plays a critical role in esthetic outcomes, because of the close relationship with the medial frontalis and the potential risk to be affected by BoNT-A injection in this region, that ultimately, can affect the interplay between the brow depressors and frontalis, which determine brow position [10]. Bertucci et al. [34] demonstrated that less favorable dynamic brow results were associated with medial corrugator injections placed above the medial brow, lateral corrugator injections performed deeply or excessively high, and procerus injections positioned superior to the inferior medial brow line. Anatomical dissections and surgical observations consistently demonstrate that the majority of the corrugator supercilii muscle is located below the superciliary arch, positioned immediately deep to the eyebrow [33].

This manuscript combines a literature review with practical clinical experience, which limits the level of evidence compared with controlled studies. The illustrative cases are intended to support the discussion and should be interpreted as hypothesis-generating rather than conclusive. On the other hand, the integration of evidence and expert clinical experience may provide practical guidance for injectors, helping them individualize patient treatment.

## 4. Conclusions

Eyebrow reshaping and repositioning with botulinum toxin type A should be performed through an individualized and anatomically guided approach rather than a standardized treatment strategy, considering eyebrow morphology, facial shape, muscular recruitment patterns, and pre-existing asymmetries. Eyebrow position and morphology are influenced by gender characteristics, aging-related changes, and, most importantly, by the dynamic balance between the frontalis muscle and the medial and lateral brow depressor muscles, requiring a comprehensive understanding of upper facial anatomy, muscular function, contraction patterns, and individual facial proportions.

Precise modulation of the frontalis and brow depressor muscles through appropriate selection of injection points, depth, dosage, and neurotoxin diffusion characteristics allows customization of eyebrow shape and position according to each patient’s esthetic needs while minimizing complications such as eyebrow ptosis, asymmetry, and an unnatural appearance.

Furthermore, upper face neurotoxin treatment extends beyond dynamic rhytid correction, representing an important tool for facial harmonization and structural balance. Maintaining the functional equilibrium between eyebrow elevator and depressor muscles remains fundamental to achieving harmonious, natural, and individualized esthetic results.

## 5. Materials and Methods

### 5.1. Search Strategy

To objectively demonstrate a systematic, customizable approach for BoNT-A injections to reshape and reposition eyebrows according to individual facial shape enhancing balance, a narrative literature search was conducted across multiple databases, including PubMed, Scielo, and the Cochrane Library. Publications from 2000 through 2026 were included to capture both pioneering research and current consensus on treatment approaches.

The search strategy employed the following terms: (eyebrow OR eyebrow shape OR eyebrow design) AND (botulinum toxin A) AND (Facial shape). To further explore esthetic eyebrow shape treatments, searches were conducted using (Eyebrow OR eyebrow shape OR eyebrow design) AND (botulinum toxin A); and (Eyebrow OR eyebrow shape OR eyebrow design) AND (Facial shape). An adjunctive search was performed focusing non IncobotulinumtoxinA, using the following terms: (eyebrow shape OR eyebrow design) AND (IncobotulinumtoxinA) AND (facial shape); (eyebrow shape) AND (IncobotulinumtoxinA); and (eyebrow) AND (IncobotulinumtoxinA). Additional relevant studies were identified through a manual review of the references cited in the included articles.

### 5.2. Eligibility Criteria

The inclusion criteria for this study included randomized controlled trials/non-randomized controlled trials [RCTs/nRCTs], single-armed studies and case series that examined the effects of BoNT-A for the treatment of upper face dynamic wrinkles that included analysis of the effect on eyebrow shape. Additionally, studies that evaluated facial shape and the ideal eyebrow shape were considered eligible for inclusion. Review articles and conference reports were excluded.

## Figures and Tables

**Table 1:** Adequate eyebrow position according to facial shape characteristics and concerns about IncoBoNT-A injection.

Facial Shape	Characteristics	Adequated Eyebrow Position	Warnings in Toxin Injection
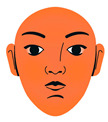 Oval	This is the ideal facial shape. Balanced proportions, with slightly wider cheekbones than the forehead and a gently rounded chin.	Curved, Sharp Angled, Soft Angled, Rounded or Flat Eyebrow plays no significant role in making the face appearance.	As this face shape is considered ideal and intrinsically balanced, toxin application can be guided by personal preferences.
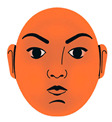 Rounded	Soft, curved lines with similar width and length of the face measures. Cheekbones and face width are almost equal, with a rounded jawline.	High Arched, Low/Medium tail, helps elongate the face’s appearance.	Rounded eyebrows can turn that same face into a “beach ball” To make a round face appear oval, one should apply lines that go up the face. The peak is best moved out toward the end of the eyebrow. This lets the lines of the brow go up and down as much as possible.
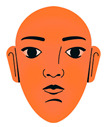 Long	Longer length than width measures, with straight cheekbones and a squared jawline.	Flat and relatively lower-posited or soft-angled eyebrows add width to the face’s appearance.	Avoid arched and picked brows, because this will create vertical lines making the face appear even longer.
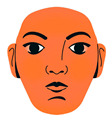 Square	Strong jawline and equal width at the forehead, cheeks, and jaw.	Strong angled eyebrows balance with the jawline, harmonizing the face’s appearance	The peak of the brow is most effective when directly above the square of the jaw.
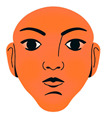 Heart	Forehead and cheekbones with the same width, a narrower jaw and more angled chin and long straight jaw lines. Longer length than width measures.	A round shape helps by adding curves to soften the face and emphasizes the lovely heart shape	A round eyebrow shape, with the pick in between the lateral limbus and lateral canthus line helps to soften and balance the face.

**Table 2:** Point of injection according to the shape of the eyebrows and how to reach the eyebrow shape result and warnings regarding application.

	**Objective to Be Reached**	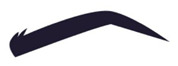 Laterally Arched	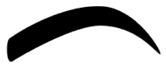 Rounded	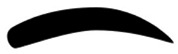 Flat
**Original** **Shape**				
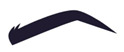 Laterally arched			Glabella + orbicularis (red, green and blue points) + frontalis (blockage partial of the mid-pupillary line compared to the outer canthus line in the intermediate and superior lines. Inferior limit line—inject only in the facial central line.	Glabella + orbicularis (only lateral points, if necessary) + frontalis (higher dosage laterally compared to the central part in the intermediate and upper lines, injecting the highest dosage in the outer canthus line. Inferior limit line—inject in the mid-pupillary line and outer canthus line, avoid injecting the central points)
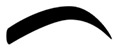 Rounded		Glabella + orbicularis (red points) + frontalis (highest dosage in the central part (including mid-pupillary line) lower dosage laterally in the intermediate and superior lines. Inferior limit line—inject in the facial central line and inner canthus line and another point using lower dosage in the mid-pupillary line.		Glabella + orbicularis (only lateral points, if necessary) + frontalis (highest dosage on the mid-pupillary line and outer canthus line in the intermediate and upper lines. Inferior limit line—inject in the mid-pupillary line and outer canthus line.
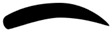 Flat		Glabella + orbicularis (red points) + frontalis (highest dosage in the central part (including mid-pupillary line) lower dosage laterally in the intermediate and superior lines. Inferior limit line—inject in the facial central line and inner canthus line and another point using lower dosage in the mid-pupillary line.	Glabella + orbicularis (red, green and blue points) + frontalis (highest dosage in the central facial line and inner canthus line, lower dosage laterally in the intermediate and superior lines. Inferior limit line—inject only in the facial central line.	

**Table 3:** Schematic of BoNT-A injection according to the muscle.

Muscle Group	Number of Injection Points	Injection Technique *	Dosage
Occipitofrontalis	Customized	Insert 30% of the needle angled upwards. Extend the injection sites laterally along the lateral canthus.	0.5 to 2 U/point
Corrugator supercilii	2 (origin and tail) * 3rd point (lateral, blending with orbicularis oculi fibers)	Origin: introduce 100% of the needle. Insertion: introduce 30% of the needle. * 3rd point: introduce the needle bevel only.	4 to 6 U/point. * 3rd point: 0.5 to 2 U
Procerus	1 to 2	Introduce 50% of the needle directed upwards.	4 to 8 U
Depressor supercilii	1	Subdermal injection, directing the needle medially and upwards.	1 to 2 U
Orbicularis oculi	3 to 8 * MPL point (blending of lateral corrugator with orbicularis oculi fibers)	Insert 30% of the needle superficially, directing the needle away from the eye. * MPL point: introduce the needle bevel only. Care should be taken to avoid injecting into the supro-orbital forâmen.	4 U/point (lateral radial lines) 2 U/point (inferior line and lateral end of the brow) 0.5 to 2 U (superior lateral orbicularis)

* Our results have been achieved with incobotulinumtoxinA.

## Data Availability

No new data was created.

## Abbreviations

The following abbreviations are used in this manuscript:

BoNT-A	Botulinum toxin type A
IncoBoNT-A	IncobotulinumtoxinA
OnaBoNT-A	OnabotulinumtoxinA
MPL	Mid-pupillary line
